# Advances in Cancer Immunotherapy in Solid Tumors

**DOI:** 10.3390/cancers8120106

**Published:** 2016-11-24

**Authors:** Smitha Menon, Sarah Shin, Grace Dy

**Affiliations:** 1 Division of Hematology and Oncology, Department of Internal Medicine, Medical College of Wisconsin, Milwaukee, WI 53226, USA; spmenon@mcw.edu; 2 Department of Internal Medicine, State University of New York at Buffalo, Buffalo, NY 14228, USA; sjshin2@buffalo.edu; 3 Department of Medicine, Roswell Park Cancer Institute, Buffalo, NY, 14263, USA

**Keywords:** immunotherapy, immune editing, tumor microenvironment, T cell exhaustion, checkpoint inhibitors, cancer vaccines, CAR T cell therapy

## Abstract

Immunotherapy is heralded as one of the most important advances in oncology. Until recently, only limited immunotherapeutic options were available in selected immunogenic cancers like melanoma and renal cell carcinomas. Nowadays, there is an improved understanding that anti-tumor immunity is controlled by a delicate balance in the tumor microenvironment between immune stimulatory and immune inhibitory pathways. Either by blocking the inhibitory pathways or stimulating the activating pathways that regulate cytotoxic lymphocytes, anti-tumor immunity can be enhanced leading to durable anti-tumor responses. Drugs which block the immune regulatory checkpoints namely the PD-1/PDL1 and CTLA 4 pathway have shown tremendous promise in a wide spectrum of solid and hematological malignancies, significantly improving overall survival in newly diagnosed and heavily pretreated patients alike. Hence there is renewed enthusiasm in the field of immune oncology with current research focused on augmenting responses to checkpoint inhibitors by combination therapy as well as studies looking at other immune modulators and adoptive T cell therapy. In this article, we highlight the key clinical advances and concepts in immunotherapy with particular emphasis on checkpoint inhibition as well as the future direction in this field.

## 1. Introduction

Our body’s immune system maintains a very sophisticated and powerful defense against “non self” antigens. For over 200 years, investigators have known that this defense by the immune system can help eradicate cancer cells but attempts to harness its potential were not very successful. Certain malignancies like renal cell cancers and melanomas were thought to be more immunogenic, as unlike other malignancies, they were resistant to conventional chemotherapy but seemed to regress with high dose cytokine therapy [1]. In order to augment tumor response, cytokines were combined with chemotherapy, so-called “chemoimmunotherapy” but this was hampered by high rates of toxicity without significant improvement in survival outcomes [2]. Interestingly, cytokine therapies provided robust benefit only in a subset of patients, mostly in those who developed clinical or serologic evidence of autoimmunity [3]. There were mixed results with other “immunomodulating” agents. Levamisole, an antihelminthic drug was found to have immune potentiation properties and was approved in colorectal cancer as an adjunct to 5 FU but later studies seemed to show no benefit [4]. Bacillus Calmette-Guerin (BCG) was developed as a vaccine against Tuberculosis but provided robust anti-tumor responses when given intravesically in bladder cancer [5]. Intravesical BCG continues to be the standard of care for superficially invasive bladder cancer since it was first approved in 1990 for this indication [6]. It is clear that while immunotherapy held a lot of promise, more progress had to be made.

There has since been a great momentum in the field of immuno-oncology after Allison and other investigators published pioneering work proving that, besides antigen presentation, a second co-stimulatory signal was essential to activate cytotoxic T cells in order to provide anti-tumor immunity [7]. In addition, inhibitory pathways, which dampen T cell activation and promote tumor growth were identified [8,9]. Blocking these pathways with monoclonal antibodies called checkpoint inhibitors has provided durable clinical responses in a variety of malignancies even in cancers like lung cancer, which were not previously thought to be immunogenic [10,11,12,13]. Furthermore, understanding of tumor immunity has evolved from a theory of Immune Surveillance to the new concept of Tumor Immune Editing. In this article, we examine recent advances in the understanding of Tumor Immunity, some of the key therapeutic innovations and the unique side effects and clinical caveats while using Immunotherapy.

## 2. Developments in Tumor Immunology

### 2.1. Concept of Immune Editing

There is now an improved understanding of the complex interaction between immune system and tumor cells.

For decades, immune surveillance was the prevailing theory in tumor immunity, which referred to the recognition of tumor cells as foreign by the immune system leading to its eradication. This has been replaced by the theory of Immune Editing put forth by Schreiber and colleagues [14] where they hypothesize that the body’s immune system interacts with the tumor in three distinct phases namely Elimination, Equilibrium and Escape. In the elimination phase, both innate and adaptive immunity work towards eliminating newly formed cancer cells. Cancer cells that may evade elimination enter the equilibrium phase where they are kept dormant by the immune cells. This eventually leads to the selection of tumor cell variants that may be less immunogenic. Finally, cancer cells enter the escape phase which is considered a hallmark of cancer where they resist immune control leading to clinically apparent disease. Thus, the immune system helps to shape or edit the tumor response to immunity indirectly promoting cancer growth.

### 2.2. Importance of the Tumor Microenvironment (TME)

We have now moved away from the simplistic view of considering tumors as a collection of cancer cells alone. It is recognized that cells adjacent to tumor cells namely stromal cells, immune cells, fibroblasts, endothelial cells all contribute to the tumor milieu and impact anti-tumor immunity. Among the immune cells, CD8+ T cells and CD4 helper T cells 1 (TH1), NK cells, M1 macrophages, dendritic cells work against the tumor while regulatory T cells (Treg), M2 macrophages, myeloid derived suppressor cells (MDSC), CD4 helper T cells 2 (TH2) cells promote tumor growth [15]. The pro-tumor immune cells in addition to the stromal and endothelial cells form an immunosuppressive network in the tumor microenvironment. The balance between pro-tumor and anti-tumor immune cells is predominantly coordinated by specific chemokines and adhesion molecules, but other factors such as tumor induced neovascularization seem to play a role in maintaining the tumor milieu. The chemokines CX3CL1, CXCL9 and CXCL10 helps to attract CD8+ T cells, memory cells and TH1 cells while CCL19, CCL17, CCL22, CXCL13 and IL-16 helps to recruit Treg cells. The immune cells can be found within the core of the tumor, in the invasive margin or within tertiary lymphoid structures (TLS) that occur within the tumor analogous to secondary follicles in lymph nodes [15]. TLS are surrounded by specialized vascular structures called high endothelial venules (HEV) which helps to recruit immune cells from the blood [16]. The distribution of the immune cells within the tumor and in the microenvironment has been termed “Immune Contexture” and certain patterns like increased density of CD8+ T cells and memory T cells have been associated with a good outcome [15]. A clear understanding of the dynamic interplay between the various cells in the TME is key to identifying new therapeutic targets.

### 2.3. Recognition of T Cell Exhaustion in the Tumor Microenvironment (TME)

CD8+ T effector cells are crucial to maintain successful adaptive immunity. They start out as naïve T cells and with antigen stimulation transform to effector T cells which produce cytokines and destroy cancer cells. Over time, some of the effector T cells undergo apoptosis and the rest will differentiate into memory T cells. There is increased recognition that CD8+ T cells are hypofunctional in the TME. In this state called T cell exhaustion, T cells express high levels of inhibitory receptors, progressively lose their ability to produce cytokines IL-2, TNF-alpha, interferon gamma and granzyme and thus are unable to effectively eliminate cancer cells [17]. T cell exhaustion was initially identified in chronic infections but is now well described in the setting of cancer. The main etiology seems to be sustained antigen stimulation [18]. Chronic exposure to antigens lead to increased expression of inhibitory receptors namely programmed cell death protein 1 (PD-1), cytotoxic T lymphocyte antigen-4 (CTLA-4), T-cell immunoglobulin domain and mucin domain protein 3 (TIM-3), lymphocyte activation gene 3 protein (LAG-3), band T lymphocyte attenuator (BTLA) and T-cell immunoglobulin and immunoreceptor tyrosine-based inhibitory motif domain (TIGIT) [17,19]. Blockade of these inhibitory receptors may partially restore T cell function improving their ability to eradicate cancer cells. The clinical success of PD-1 inhibitors and anti CTLA-4 antibodies has validated this concept. However, combination approaches targeting multiple inhibitory receptors may be needed for more efficient restoration of T cell function but has to be balanced against increased cytokine release and autoimmune reactions from excessive T cell function.

## 3. Therapeutic Advances

### 3.1. Checkpoint Inhibitors

The two major classes of checkpoint inhibitors currently used in the clinic are anti CTLA-4 antibodies (e.g., Ipilimumab) and anti PD-1 (Nivolumab, and Pembrolizumab) antibodies. They are approved by the United States Food and Drug Administration (FDA) in various malignancies as discussed below.

Antibodies against the PD-L1 ligand are also in late-phase clinical development namely Durvalumab, Avelumab and Atezolizumab. Table 1 lists additional examples of investigational checkpoint inhibitors in late preclinical/early clinical trial testing.

### 3.2. Mechanism of Action

CTLA-4 is an inhibitory molecule present on T cells, which is homologous to the CD28 receptor. The interaction between CD28 on T cells and the B7 receptor (CD80 or CD86) on antigen presenting cells provides the second “co-stimulatory signal” which is essential for T cell priming, which is the differentiation of naïve T cells into activated T cells. CTLA-4 has higher affinity for B7 compared to CD28 thus competitively binding to the B7 receptors interrupting the co-stimulatory signals necessary for T cell priming. By blocking CTLA-4, the inhibitory effect on the priming phase is released leading to unrestricted T cell activation [20]. In contrast, PD-1 is a receptor that is expressed in activated T cells and the interaction between PD-1 and its ligands PD-L1 and PD-L2 is an innate mechanism to reduce auto immunity and promote tolerance. PD-L1 is more extensively expressed than PD-L2 with upregulation in lymphoid cells, epithelial cells, endothelial cells, fibroblasts while PD-L2 is expressed mainly by dendritic cells, lung epithelial cells and macrophages [21], This pathway is exploited by the cancer cells which express PD-L1 and their interaction with PD-1 positive tumor infiltrating lymphocytes helps to dampen the anti tumor response. By blocking the PD-1/PD-L1 interaction either by anti PD-1 or PD-L1 antibodies, antitumor immunity can be restored [22]. In addition, there is some evidence that PD-1/PD-L1 interaction is key to the maintenance of Treg cells [23] and thus blockade of this pathway may boost anti-tumor immunity in the tumor micro environment by affecting different cell types.

In summary, anti CTLA-4 and anti PD-1 agents have distinct mechanisms of action. CTLA-4 inhibition occurs during the priming phase while PD-1 blockade occurs in the effector phase in local tumor tissues [24]. CTLA-4 inhibition may affect broader T cell function and this may explain why anti CTLA-4 antibodies are associated with higher grade 3 immune side effects when compared to PD-1 inhibitors.

## 4. Clinical Trials

### 4.1. Melanoma

Melanoma has been at the forefront of recently successful immunotherapy trials. Ipilimumab, the anti-CTLA-4 antibody, was the first to be studied but with the advent of the PD-1 inhibitors which have a better toxicity and response profile, its use as a single agent has decreased in the clinic. In the pivotal phase 3 trial, 676 previously treated patients with metastatic melanoma were randomized to Ipilimumab (*n* = 137), vs. Ipilimumab plus glycoprotein 100 vaccine (*n* = 403), or glycoprotein 100 vaccine alone (*n* = 136) with increased median survival rates in patients receiving Ipilimumab plus gp 100 compared to gp 100, 10 months vs. 6.4 months (HR 0.68 *p* < 0.001) [13]. There was no difference between the two ipilimumab groups suggesting that gp 100 did not provide an additive effect. Ipilimumab in combination with dacarbazine was found to be superior to dacarbazine alone in patients with previously untreated melanoma with an overall survival (OS) of 11.2 months vs. 9.1 months with higher rates of grade 3 or 4 adverse events (56.3% vs. 27.5% *p* < 0.001) [25].

In the KEYNOTE 006 trial, pembrolizumab was investigated at two dose schedules, 10 mg/kg every two weeks vs. every three weeks, and was compared to Ipilimumab at 3 mg/kg for four doses. Treatment with pembrolizumab was superior at both dose schedules compared to Ipilimumab with increased response rates 33.7% vs. 32.9% vs. 11.9% respectively. The one year survival rates in the pembrolizumab two-week arm and three-week arm were improved at 74.1% and 68.4% compared to 58.2% in the Ipilimumab arm [26]. Serious adverse events were more common with Ipilimumab 20% vs. 13/10% compared to the pembrolizumab arms. Similarly, nivolumab showed superior OS and objective response rate (ORR) compared to dacarbazine in previously untreated patients with BRAF wildtype metastatic melanoma in the CheckMate 066 phase III study, with one-year survival rate of 73% and ORR of 40% seen with nivolumab [27]. These pivotal trials led to the widespread adoption of anti-PD1 agents as first-line therapy in melanoma.

#### 4.1.1. Combination Approaches

The combination of nivolumab and ipilimumab has shown significant activity and is currently approved in the United States for the first line treatment of advanced BRAF negative melanoma. In the Checkmate 067 study, 945 untreated patients with advanced melanoma underwent a 1:1:1 randomization to either nivolumab vs. combination nivolumab and ipilimumab vs. ipilimumab. The primary endpoints of the study were progression-free survival (PFS) and OS. At a median follow up of 12 months, the PFS in the combination arm was superior (11.5 months), compared to nivolumab (6.9 months) and ipilimumab (2.9 months) [28]. The combination arm had higher response rates (58% vs. 44% vs. 19%) but also had much greater grade 3 or 4 adverse events at 55% (combination) vs. 16% (nivolumab) vs. 27% (ipilimumab). The benefit with the combination seemed to be more evident in PD-L1 negative patients. The median PFS in the PD-L1 negative patients in the combination, nivolumab and ipilimumab were 11, 5 and 3 months, respectively, vs. 14, 14 and 4 months in the PD-L1 positive patients. An update of this trial was presented at the 2016 American Society of Clinical Oncology (ASCO) annual meeting, where after more than 18 months of follow up, the combination continue to outperform the single agent arms. The benefit seemed to persist regardless of PD-L1 or BRAF mutation status [29]. Keynote-029 is a study examining combination pembrolizumab and ipilimumab with preliminary results showing high activity for this combination with PFS of 70% at six months but with 25% grade 3 or 4 toxicities [30].

#### 4.1.2. Checkpoint Inhibition as an Adjuvant Strategy

Ipilimumab is approved for adjuvant treatment of high risk melanoma based on the results of a European Organization for Research and Treatment of Cancer (EORTC) 18071 trial which showed improved relapse free survival (RFS) compared to placebo, with three-year RFS of 46.5% vs. 34.8%, HR (0.75, 95% CI 0.64–0.9) [31]. Ipilimumab was given at a higher dose of 10 mg/kg compared to 3 mg/kg used in the metastatic setting with four doses given every three weeks followed by treatments every three months for three years. Ninety percent of patients had immune side effects with 36.5% having grade 3 events and only 29% completed more than one year of treatment. An ongoing ECOG study 1609 (NCT01274338) is comparing ipilimumab at 10 mg/kg dose versus 3 mg/kg versus high dose interferon and may clarify the use of ipilimumab in this setting. PD-1 inhibitors namely nivolumab and pembrolizumab are also being studied as adjuvant therapy in phase III studies (NCT02388906, NCT02362594, S1404) and results are awaited.

### 4.2. Lung Cancer

#### 4.2.1. Non-Small Cell Lung Cancer (NSCLC)

Recent advances in checkpoint inhibition have provided new treatment options for patients with advanced NSCLC, including those whose tumors have failed first line therapy. In a Phase III clinical trial CheckMate 017, 272 patients with advanced squamous NSCLC, were randomized to receive antiPD-1 blockade with nivolumab (*n* = 135) or docetaxel chemotherapy (*n* = 137). The nivolumab arm had greater median OS—9.2 months vs. 6.0 months (HR 0.59, *p* < 0.001) and a higher percentage of 1-year survival—42% vs. 24%—than second line docetaxel). PFS with nivolumab was 3.5 months vs. 2.8 months in the docetaxel arm (HR 0.62, *p* < 0.001). Importantly, the ORR was 20% on nivolumab compared to 9% with docetaxel and fewer patients reported treatment-related adverse effects of grade 3–4 on nivolumab (7% vs. 55%) [10]. Additionally, PD-L1 expression did not influence survival benefit. Nivolumab similarly outperformed docetaxel in a phase III clinical trial among 582 patients with advanced non-squamous NSCLC (CheckMate 057) receiving second line therapy. There was significant improvement in median OS (12.2 months vs. 9.4 months, HR 0.73, *p* = 0.002), 12-month OS (51% vs. 39%) and 18-month OS (39% vs. 23%) survival, and ORR (19% to 8%) [11] in the nivolumab arm compared to docetaxel. Survival in the PD-L1 negative patients receiving nivolumab was similar to patients receiving docetaxel. However, in contrast to the squamous lung cancer patients in Checkmate 017, PD-L1 positive status seemed to predict for response and improved survival in non squamous patients. Such studies highlight the improvements targeted therapy against PD-1 can provide, even in the setting of refractory disease. 

Another therapeutic agent that targets the PD1/PD-L1 pathway with activity in NSCLC is pembrolizumab. A phase 1 study that was a part of the larger KEYNOTE-001 trial investigated the side effects, safety, and anti-tumor activities of pembrolizumab in advanced NSCLC. ORR was seen in 19.4% of patients with median duration of response of 12.5 months. Median duration of PFS was 3.7 months and median duration of OS was 12.2 months in patients treated with pembrolizumab. Importantly, efficacy of the therapeutic agent differed depending on the tumoral expression of PD-L1—the median duration of PFS in those with at least 50% expression compared to those with 1%–49% was significantly different at 12.5 months and 7.2 months, respectively. Grade 3 or 4 adverse events were seen in 9% of patients with the most common side effects being fatigue, pruritus, and decreased appetite. The results of this study led to the accelerated approval of pembrolizumab in patients with PD-L1 expressing NSCLC upon disease progression after other therapies [12]. Subsequent phase 2/3 randomized controlled clinical trial investigating pembrolizumab versus docetaxel in previously-treated, PD-L1 positive advanced NSCLC demonstrated superiority of pembrolizumab compared to chemotherapy in this population [32].

With their role now firmly established as a therapeutic option in NSCLC, these agents are currently being evaluated in the first-line setting. Current efforts are focused on establishing these agents as first line therapy in various phase 3 studies, either as monotherapy in PD-L1 positive NSCLC (CheckMate 026 NCT02041533, KEYNOTE-024 NCT02142738, MYSTIC NCT02453282) or in combination with ipilimumab or chemotherapy (CheckMate 227 NCT02477826, KEYNOTE-189 NCT02578680, KEYNOTE-407 NCT02775435, NEPTUNE NCT02542293, Impower 132 NCT02657434, Impower 150 NCT02366143). The results from KEYNOTE-024 were recently published showing a significant advantage with first line pembrolizumab in patients with increased PD-L1 activity defined as expression in more than 50% of tumor cells. The median PFS in patients receiving pembrolizumab was 10.3 months vs. 6.0 months in the chemotherapy group, HR 0.5; 95% CI, 0.37 to 0.68 *p* < 0.001. At six months, 80.2% of patients in the pembrolizumab group were alive compared to 72.4% in the chemotherapy group (HR for death, 0.60; 95% CI, 0.41 to 0.89; *p* = 0.005) Pembrolizumab was associated with higher response rates (44.8% vs. 27.8%) and the responses were very durable with median duration not reached in the immunotherapy group (range 1.9+ to 14.5+ months) vs. 6.3 months in the chemotherapy group. First line pembrolizumab was well tolerated, with only 26.6% patients having grade 3 or higher treatment related adverse events compared to 53.3% in the chemotherapy group [33]. These results are practice changing and Pembrolizumab has received US FDA approval for first line therapy of metastatic non small cell lung cancer patients with high PD-L1. Beyond first line-therapy for metastatic disease, various clinical trials currently are also exploring its use in other settings, such as consolidation therapy after concurrent chemoradiation in locally advanced NSCLC (PACIFIC NCT02125461) or as adjuvant therapy in resected NSCLC. The ANVIL and PEARLS studies are two phase 3 clinical trials that are currently exploring anti-PD-1/PD-L1 targeted agents for the treatment of NSCLC. ANVIL study (NCT02595944) will evaluate adjuvant nivolumab versus observation in resected lung cancer and its impact on disease-free survival and OS. The PEARLS study investigates pembrolizumab versus placebo in resected NSCLC with or without standard adjuvant therapy with primary outcome of disease-free survival and secondary outcomes of OS and lung cancer specific survival (NCT02504372). Immunotherapy with checkpoint inhibitors marks a new era in the evolution of systemic treatment approaches for NSCLC.

#### 4.2.2. Small Cell Lung Cancer (SCLC)

In contrast to NSCLC, the role of PD-1 inhibition in the treatment of SCLC is still under investigation. In the KEYNOTE-028 Phase Ib study or pembrolizumab in patients with PD-L1positive solid tumors, only 42 of 147 SCLC patients (28.6%) were found to have tumoral PD-L1 expression as determined by immunohistochemistry. Of these patients, 20 were treated with pembrolizumab with seven patients (35% ORR; 95% CI 15%–59%) exhibiting partial response [12]. Importantly, safety profile was in line with those previously documented for other tumor types. Similarly, CheckMate 032 was a phase I/II study designed to assess the response to nivolumab with or without ipilimumab in previously-treated advanced SCLC regardless of PD-L1 expression. ORR was 10% and 23%/19% in the single and two combination treatment arms evaluating different dose schedule, respectively. Toxicities were expectedly higher with nivolumab+ipilimumab combination [34]. These agents are being evaluated in various phase II/III trials, such as in combination with first-line chemotherapy (EORTC REACTION trial NCT02580994), as maintenance first-line treatment (CheckMate 451 NCT02538666, NCT02359019) or in the relapsed setting (CheckMate331 NCT02481830).

### 4.3. Genitourinary (GU) Cancers

The experience with renal cell carcinoma further highlights the benefits of these agents. When compared to the mTOR inhibitor everolimus, blockade of PD-1 signaling with nivolumab in the phase 3 CheckMate 025 increased median OS (25.0 months vs. 19.6 months), decreased hazard ratio (0.73; *p* = 0.002), and increased ORR (25% vs. 5%; *p* < 0.001) in patients who had received prior anti-angiogenic therapy [35]. These results led to the approval of nivolumab in November 2015 for this patient population. Indication in the first-line setting with anti-PD1 agents in combination with antiangiogenic agents or ipilimumab is being evaluated in various phase 3 trials (NCT02811861, CheckMate 214 NCT02231749, JAVELIN Renal 101 NCT02684006, NCT02420821).

The use of checkpoint inhibitors in other GU cancers has also shown promising efficacy. A phase Ia study with atezolimumab, demonstrated durable activity in previously treated metastatic urothelial bladder cancer with greater responses seen in patients with higher PD-L1 expression in tumor-infiltrating immune cells (IC) [36]. Furthermore, a phase 2 international, multi-center, single-arm trial investigating the safety and efficacy of atezolimumab in locally advanced or metastatic urothelial cancer patients during or following platinum-based therapy demonstrated durability and tolerability of this drug with ORR of 26% in patients with ≥5% PD-L1 expressing IC in their tumors, with ongoing response at median follow up at 11.7 months in 84% of responders. Grade 3/4 adverse events were seen in 15% of treated patients with no treatment-related deaths [37]. Based on these results, atezolimumab received US FDA approval for this patient population. Additional data from recently completed or ongoing phase 3 trials with either anti-PD-1 monotherapy or in combination regimens (IMvigor 2011 NCT02302807, JAVELIN Bladder 100 NCT02603432, NCT02516241), which may help further elucidate the utility of these therapeutic agents in urothelial cancers.

### 4.4. Malignant Mesothelioma

Similarly, immunotherapies were evaluated in mesothelioma given positive response in other tumor types. An open label, single arm phase 2 clinical trial (MESOT-TREM 2008), evaluated the safety and efficacy of tremelimumab, an anti-CTLA4 antibody, in unresectable malignant mesothelioma. Tremelimumab demonstrated no complete remission, but had partial response in 6.9% and stable disease in 24.1%, demonstrating disease control in 31% of patients [38]. This led to the design of the randomized placebo-controlled phase III DETERMINE trial evaluating tremelimumab in patients with unresectable pleural or peritoneal malignant mesothelioma as second or third-line therapy. Unfortunately, this study did not achieve the primary endpoint of OS superiority compared to placebo [39]. In contrast, subset analysis of 25 patients enrolled in KEYNOTE 028 clinical trial with malignant pleural mesothelioma demonstrated 28% ORR and 48% disease stability with tolerable adverse effects, mostly grade 1 with rash [40]. As with other solid tumors, further investigation of PD1 agents either as monotherapy or in combination strategies are ongoing (NCT02588131, NCT02716272).

### 4.5. Merkel Cell

Advanced Merkel cell carcinoma is considered an aggressive type of immunogenic skin cancer with a median progression-free survival of three months on cytotoxic chemotherapy. Checkpoint inhibitors have also become of particular interest as first-line therapy against Merkel cell carcinoma due to the elevated levels of PD-L1 expression on these tumor cells. A phase 2, multicenter study with 26 stage IIIb–IV Merkel cell carcinoma patients was conducted to assess the efficacy of pembrolizumab in systemic chemotherapy-naïve patients with Merkel cell carcinoma. Complete response was observed in four patients with 10 others exhibiting partial response for a total of 56% overall response rate (95% CI: 35%–76%). Response duration ranged from 2.2 months to 9.7 months, with a mean progression-free survival of six months. Importantly, PD-L1 expression was more frequent in tumors positive for Merkel cell polyomavirus (MCPyV) with 71% compared to 25% among non-virus associated Merkel cell carcinomas; the response rate among PD-L1+ tumor bearing patients were only minimally higher at 62% (44% among MCPyV negative patients). As seen with other tumor types, the frequency of severe grade 3 or 4 treatment-related adverse events was low (15%)—two patients with grade 4 adverse events demonstrated myocarditis and two others had elevated aminotransferases. Further studies comprised of larger cohorts and more robust longitudinal observations are necessary and are currently ongoing (NCT02488759, NCT02267603, JAVELIN Merkel 200 NCT02155647) [41].

### 4.6. Colorectal Cancer

PD-1 blockade immunotherapy has been said to be most effective in tumor types that have high amounts of somatic mutations. A Phase II trial characterizing the role of anti-PD1 immunotherapy in patients with colorectal cancers with high microsatellite instability (MSI) found that patients with colorectal cancers that have deficient mismatch repair mechanism had significantly improved responses (40%) and immune-related progression-free survival rates (78%) than those who had cancers with an intact mismatch repair (0% and 11% respectively). These results demonstrated a hazard ratio of 0.10 for disease progression or death with *p* < 0.001 for PD-1 blockade in mismatch repair deficient colorectal cancer. The difference in survival was demonstrated to not be an effect of prognostic differences from the time of metastatic diagnosis. Of note, all of the patients with mismatch repair deficient tumors not associated with Lynch syndrome had an objective response, while those associated with Lynch syndrome exhibited variable responses (3 out of 11 patients). The study also found that genomic analysis of tumors without mismatch repair demonstrated a mean of 1782 somatic mutations, while the proficient tumors only averaged 73 mutations [42] Importantly, membranous PD-L1 expression was only observed in patients with mismatch repair deficient cancers. These findings reveal the importance of anti-PD1 immunotherapy not only for disruption of the PD1 signaling cascade, but also the ability of this inhibition to prime the immune system to response robustly to highly mutagenic cancers that may be expressing neoantigens.

### 4.7. Squamous Cell Cancers

Despite being the 5th most common cancer worldwide and a first-line chemotherapeutic regimen including platinum, taxanes, cetuximab, 5-FU, and methotrexate, patients only gain approximately 6–10 months of median overall survival post-intervention. A phase 1b, multi-cohort trial of 132 patients with recurrent or metastatic HNSCC, regardless of HPV or PD-L1 status, and measurable disease treated with pembrolizumab for 24 months found that 24.8% demonstrated overall response to therapy, of which 0.9% had complete response and 23.9% had partial response. When differentiated further depending on HPV status, of the 34 HPV+ patients, 2.9% had complete response and 17.6% had partial response with 20.6% overall response. The 81 HPV-patients demonstrated no complete response but 27.2% partial response. Fifty-nine percent of the participants experienced decrease in the target lesion with median time to response being nine weeks. Eighty-six percent of responding patients remain on therapy. Of note, the majority of the patients in the study had previously failed treatment –22.7% had one prior line of therapy, 21.2% with two prior lines of therapy, and 37.9% had three or more prior lines of therapy; only 16.7% had no prior treatment for their metastatic or recurrent cancer [43]. While this large immunotherapy study demonstrated activity of pembrolizumab in both HPV positive and negative populations and in heavily pre-treated population, further studies are underway assessing the utility of anti-PD1 agents, either singly or in combination, in the setting of recurrent and metastatic HNSCC (CheckMate 651 NCT02741570, KEYNOTE 048 NCT02358031).

Checkpoint inhibitors have similarly demonstrated promising efficacy in other HPV-implicated cancers, such as cervical and anal carcinomas. In the KEYNOTE-028 study, ORR to pembrolizumab was 12.5% [44]. Nivolumab, on the other hand, demonstrated ORR of 24% in patients with previously-treated metastatic anal cancer [45]. Confirmatory trials are ongoing (KEYNOTE-158 NCT02628067, CheckMate 358 NCT02488759).

### 4.8. Hepatocellular Carcinoma (HCC)

HCC is the second most common cause of cancer worldwide but has very limited systemic treatment options. Sorafenib, a multi targeted tyrosine kinase inhibitor is the current first line option in advanced HCC, conferring a median survival of 6.5 to 10 months [46,47]. HCC is thought to be have a immunosuppressive tumor microenvironment with chronic inflammation from chronic hepatitis or non alcoholic steatohepatitis leading to T cell exhaustion [48]. PD-L1 seems to be upregulated in patients with HCC and chronic hepatitis B [49]. Thus, clinical trials examining checkpoint inhibition in HCC are very promising and is currently underway [48]. A Phase 1 dose escalation study with nivolumab enrolled 47 HCC patients, but only 17 remained on the study—2 patients discontinued after complete response, 26 stopped due to progressive disease, and two were limited by adverse effects. Of those 17 patients who remained in the study, two demonstrated complete response and six had partial response. Seven out of the eight patients responded within the first three months of initiation with durable dose response across all doses and etiologies (uninfected vs. Hep C vs. Hep B). Of 42 patients evaluable for response, ORR of 19% was seen. OS at nine months was 70% and at 12 months was 62% [50]. Given these results, the study demonstrated promising durability and overall survival that warrants further studies with nivolumab in patients with advanced HCC refractory to first-line therapy with sorafenib.

### 4.9. Hodgkin’s Lymphoma

Hodgkin’s Lymphoma demonstrates major dysregulation of PD-1 ligand, which allows for the tumors to enlist the immunogenic characteristic to evade immune response. The amplification of chromosome 9p24.1 increases the expression of JAK2, which increases PD-1 ligand expression. Additionally, EBV infection associated with Hodgkin’s increases the expression of PD-1 ligand. Fortunately, this overexpression of PD-1 ligand enhances the vulnerability of Hodgkin’s Lymophoma to PD-1 ligand targeted blockade. In a phase 1 trial of nivolumab in 23 patients with Hodgkin’s Lymphoma who demonstrated refractory disease to at least one prior line of therapy (78% had prior therapy of brentuximab vendotin, 78% had autologous stem-cell transplantation, 83% had prior radiotherapy, 87% had ABVD therapy), ORR with single-agent nivolumab was 87%. Of the 20 patients with response, 60% had response by eight weeks. Median OS was not reached with median follow up duration of 40 weeks. Importantly, FISH analysis of the tumor cells in a subgroup of patients with available tumor specimen demonstrated that Reed-Sternberg cells showed 3–15 copies of the PDL1 and PDL2 genes, correlating response to amplification, copy number gain or polysomy of the immunotherapy target. Unfortunately, 52% reported grade 3–4 adverse events, including myelodysplastic syndrome, pancreatitis, pneumonitis, stomatitis, colitis, GI inflammation, thrombocytopenia, and leucopenia. No treatment-related deaths occurred [51]. Nonetheless, in the pooled analysis of the results from this study as well as another single-arm, multicenter trial, ORR and treatment benefit was similarly confirmed and thus nivolumab received accelerated approval as treatment for patients with classical Hodgkin’s lymphoma refractory to prior treatment with brentuximab and/or autologous stem cell transplantation.

## 5. Distinct Feature of Tumor Response to Checkpoint Inhibitors

There are unique caveats while assessing response to checkpoint inhibitors. While a large proportion of tumor responses occur early, similar to what can be achieved with cytotoxic chemotherapy, in a fraction of cases, there may be radiologic changes that conventionally signify disease progression (e.g., appearance of new lesions or increase in size of existing lesion) during the initial tumor assessment but subsequent imaging studies will show eventual response at a later time point. This phenomenon is called pseudo progression. This provided the impetus to formulate a new set of criteria called immune response criteria to be used in evaluating response to immunotherapeutic agents rather than the standard RECIST criteria [52]. With immune response criteria, immunotherapy is discontinued only after a confirmatory scan at least four weeks after the initial scan showing progression excludes pseudoprogression. Another distinct feature of immunotherapy is that many of the tumor responses are durable and can last years even after cessation of therapy. Additionally, aside from the known association between development of vitiligo and outcomes to therapy in melanoma patients [53], there appears to be no robust association between the development of immune-related toxicity in general with anti-tumor response, in contrast with the general experience with targeted therapies or cytotoxic chemotherapy.

### 5.1. Predictors of Response

The response to checkpoint inhibitors has not been universal. Only a fraction of the patients undergoing PD-1 therapy in lung cancer respond to treatment. The presence of PDL1 antigen by IHC staining is to date the best characterized in the clinic as a biomarker to predict treatment benefiting certain tumor types, such as nonsquamous NSCLC [11]. However, this has not been completely predictive in other studies, as exemplified by the substantial clinical activity in PD-L1 negative melanoma. Factors that may explain the variability among studies could be the use of fresh vs. archival tissue, different antibodies, different cutoffs for positivity, and tumor heterogeneity. Regardless, there is a subset of patients who are PD-L1 negative who seem to respond to PD-1 therapy and in contrast, patients who are PD-L1 positive who do not respond to therapy suggesting that other factors may be involved in response to these agents. Smoking status has been correlated with response in several studies. Smokers have increased mutational burden and a distinct mutational spectrum when compared to nonsmokers [54]. In a landmark paper, Rizvi et al. studied mutational burden in two cohorts of lung cancer patients receiving pembrolizumab and found that the increased burden of nonsynonymous mutations seem to correlate with durable clinical benefit (DCB) defined as partial response or stable response lasting >6 months [55]. High burden was defined as mutational load above the median burden of the cohort (209). In the group with the high burden of mutations, DCB was noted in 73% compared to 13% with low burden. (Fisher’s exact *p* = 0.04). Additionally, objective response rate (ORR) and progression-free survival (PFS) favored the high mutational burden group, ORR 63% versus 0%, *p* = 0.03; median PFS 14.5 versus 3.7 months, *p* = 0.01; hazard ratio (HR) 0.19, 95% confidence interval (CI) 0.05 to 0.70. Furthermore, they developed a specific molecular signature related to smoking which seems to predict for clinical benefit. The presence of neoantigens and certain DNA repair genes also seemed to correlate with benefit [55]. While the molecular smoking signature correlated efficacy, interestingly, patient self-reported smoking history did not correlate with the mutational burden. Similarly, it has been shown that efficacy of anti CTLA4 antibodies in melanoma is linked to the mutational burden and the presence of neoantigens [56]. Additionally, responses to PD-1 inhibitors are robust in mismatch repair deficient tumors, which have increased number of neoantigens [42]. Hence, evaluating the mutational burden and presence of neoantigens are crucial in determining the efficacy of immunotherapy.

There is also widespread recognition that the phenotype with T-cell-inflamed TME carries, not only prognostic implications for early stage cancers but also potentially an association with response to immunotherapies in the metastatic setting. Indeed, inclusion of inflammatory cells in IHC scoring of PD-L1 status improves the ability to predict treatment response compared to that derived from PD-L1 expression in tumor cells alone [57]. Additionally, a candidate IFNγ-gene signature including six candidate genes IDO1, CXCL10, CXCL9, HLA-DRA, STAT1 and IFN gamma developed from RNA extracted from formalin fixed paraffin embedded slides (FFPE), was also associated with ORR, PFS and OS to Pembrolizumab in head and neck, gastric and esophageal cancer [57,58]. More recently, structural variants in the 3’untranslated region of the PD-L1 were described as a potential genetic marker of response to anti-PD1 checkpoint inhibitor therapy [59] These structural variants affected multiple cancer types, including adult T-cell leukemia/lymphoma (27%), diffuse large B-cell lymphoma (8%), gastric adenocarcinoma (2%). These variants result in disruption of the 3’region of the PD-L1 gene that result in marked PD-L1 overexpression [59]. Further validation and development of these genetic signatures may help us to rationally plan therapy options for patients. Additionally, immunosequencing is a promising strategy where B or T cell receptor sequences are analyzed either from tissue or blood allowing efficient prediction of response to therapy based on presence or absence of clonal expansion [60]. Many other innovative high throughput technologies including whole exome sequencing, protein microarray, mass cytometry have shown great potential and are being evaluated [60].

### 5.2. Toxicities of Checkpoint Inhibitors

Checkpoint inhibitors are associated with a unique set of side effects called Immune related adverse events. These seem more prevalent with anti CTLA4 antibodies compared to anti PD-1/PDL1 agents as the former act in the antigen priming phase. All these agents can trigger a variety of autoimmune reactions commonly manifested as rash, colitis, hepatitis, endocrinopathies like hypothyroidism, panhypopituitarism, adrenal insufficiency etc. In a pooled analysis of 576 melanoma patients getting Nivolumab, median time of onset of skin adverse events (AE) was five weeks compared to 15 weeks in patients getting renal AEs [61]. The general approach to management of these disorders (except for hypothyroidism where thyroid replacement is started) is to hold the drug for mild to moderate events. For severe grade 3 reaction, in addition to drug discontinuation, steroids equivalent to 1–2 mg/kg is given. If there is non-resolution of symptoms, infliximab can be given, except in autoimmune hepatitis where mycophenolate mofetil is recommended [62,63]. Hepatitis may resolve within three weeks but skin AEs can take longer, up to 29 weeks to resolve.

There is clinical concern that steroids can affect the efficacy of immunotherapy. However, there may be evidence contrary to this. A retrospective evaluation of a cohort of 298 patients who received Ipilimumab showed that majority of the patients (254 patients/85%) developed immune related adverse events (IrAEs). Out of this, 35% of patients needed steroids for resolution of symptoms and in 10% of patients anti tumor necrosis factor antibody was used. The survival and time to treatment failure was similar in patients receiving steroids when compared to patients not receiving steroids [64]. In the previously described pooled analysis of patients getting Nivolumab, the response rates and duration of response was not affected in patients getting immunosuppressive therapy.

## 6. Approaches to Augment the Immune Response

### 6.1. Combinations of Checkpoint Inhibitory Receptor Antagonists or Activating Receptor Agonists

Intuitively, empiric evidence across disciplines provide rationale for approaches to augment the immune response either homologously through combinations of multiple inhibitory receptor antagonists to mitigate linear “escape” mechanisms or heterologously through combinations of checkpoint inhibitory receptor antagonist and activating receptor agonist to enhance T cell cytotoxic response bidirectionally. The well-known combination strategy of anti-PD-1/PD-L1 agents with anti-CTLA4 blockade as described previously has the most traction and clinical evidence to date. Other known checkpoint inhibitor receptors, such as Lymphocyte activation gene-3 (LAG3) and T cell immunoglobulin and mucin domain 3(TIM-3), have demonstrated preclinical synergistic activity with anti-PD1 agents and are thus being evaluated in several combination clinical trials [65,66]. Combination with co-stimulatory receptor agonists, such as with OX40 and 4-1BB, have shown preliminary results of tolerability of this approach with toxicities as expected with anti-PD1 monotherapy [67,68]. Such initial demonstration of safety is critical, given known life-threatening complications of systemic inflammatory and immune reaction, as first encountered in the development of an anti-CD28 superagonist antibody TGN1412 [69].

### 6.2. Combinations of Checkpoint Inhibitors with Chemotherapy and/or Radiation

In general, chemotherapy is considered immunosuppressive but there is evidence that chemotherapy can promote immune responses [70]. This has led to studies looking at combination of chemotherapy with checkpoint inhibition. In the phase 1 checkmate 012 study, 56 patients received doublet platinum chemotherapy with either cisplatin/pemetred, cisplatin/gemcitabine or carboplatin/paclitaxel in combination with nivolumab. As expected, there were adverse events leading to discontinuatuion in 21% of patients and 7% of patients had pneumonitis. There was promising clinical activity seen with the combination with response rates ranging from 33% to 47% across arms. Particularly, the carboplatin paclitaexl in comibnation with nivolumab 5 mg/kg had a 2-year OS rate of 62% which was very encouraging and further studies are warranted [71].

Radiation can lead to tumor shrinkage outside the radiation field, the so-called “abscopal effect” which is thought to be from the activation of the immune system by tumor specific antigens released by malignant cells killed by radiation [72]. There is preclinical data that radiation may boost immune effects by enhancing T cell function and level of PD-L1 expression and it is postulated that combining radiation with PD-1 blockade may be synergistic. [73] There are currently several ongoing studies that are evaluating the benefit of combining radiation and immunotherapy, and these will likely give us more definite evidence.

### 6.3. Modulating Extracellular Mechanisms of Immunesuppression within the TME

Indoleamine 2,3 dioxygenase 1 is an enzyme [74] that breaks down tryprophan into kynurenine. Depletion of tryptophan can lead to a immunosuppressive TME by inhibiting CD8 T cell activity, promoting regulatory T cells and decreasing expression of T cell receptors. Promising results have been seen in mice models when anti CTLA 4, PD-1/PDL1 inhibitors or GITR agonists were combined with IDO inhibitors [75]. Adenosine produced within the hypoxic TME has an inhibitory effect on T cells through signaling mediated by high-affinity A2a receptors, also expressed on a variety of immune cell subsets, such as natural killer (NK) cells and myeloid cells, and endothelial cells [76]. Currently there are a number of active clinical trials looking at the combination of checkpoint inhibitors with IDO1 inhibitors or A2a receptor antagonists and results are awaited.

NK cells are responsible for innate immunity. Killer Immunoglobulin-like receptors (KIRs) are expressed by NK cells and they bind to MHC class 1 molecules which serves to dampen NK cell activity as a way to mitigate immune responses against normal cells which generally express high levels of MHC Class I molecules [76]. Cancer cells generally retain the expression of MHC Class I molecules, hence the ability to evade NK cell-mediated cytotoxicity [77,78]. Blockade of KIR boosts NK-mediated killing of tumor cells and thus provide rationale for further clinical evaluation [79]. Eliminating or altering the function of tumor-infiltrating MDSCs via blockade of relevant signaling pathways such as CSF-1R, can potentiate the antitumor efficacy of checkpoint inhibitors [80]. CCR4, is a chemokine receptor preferentially expressed by TH2 and Treg cells that promotes recruitment of immunosuppressive cells. Mogamulizumab is a monoclonal antibody targeting CCR4 which has shown activity preclinically as well as clinically against T cell lymphoma and other lymphoproliferative diseases [81,82].

### 6.4. Modulating Intracellular Mechanisms of Immunesuppression within the TME

Bruton Tyrosine Kinase (BTK) is a non-receptor tyrosine kinase, which plays a very crucial role in the B cell receptor signaling pathway. Ibrutinib is a non reversible inhibitor with excellent clinical activity in B cell malignancies [83]. In addition, Ibrutinib blocks ITK1 (interleukin-2-inducible kinase) a key enzyme in TH2 activity helping to tilt the balance towards the T helper 1 subset thus enhancing anti-tumor immunity. It is postulated that combination PD-1 and BTK inhibition may be a promising way to improve efficacy of these agents and studies are ongoing [84]. Another target for drug therapy is the inhibition of JAK/STAT signaling in tumor cells which have been shown to confer increased susceptibility to NK cell lysis, to a similar degree seen with PD-L1 blockade [85].

## 7. Augmenting Interaction between Effector Cells and Tumor Cells

### 7.1. Bi-Specific Tcell Engager (BiTE) Antibody Technology

These are unique monoclonal antibodies which are designed to induce cytotoxic T cell response to tumor antigens independently of antigen presenting cells and major histocompatibility complex class I (MHC) molecules. BiTE antibodies have two single chain variable fragments (scFv) giving them dual specificity, one to CD3ε, a key part of T cell receptor complex crucial in the activation of T cells and the other to the tumor antigen of choice. This allows the BiTe antibody to engineer a synapse involving tumor cells and T cells allowing T cells to destroy the cancer cells [86]. The most well evaluated BiTE antibody is Blinatumomab developed against CD19 a common antigen in B cell malignancies which showed promising results in relapsed/refractory B-precursor ALL and is currently approved for this indication [87]. The challenges with this treatment is the very short half life requiring continuous infusion as well as neurological events like seizures, confusion or encephalopathy from irritation of the CNS. These CNS events are transient, reversible and are prevented by pre medication with dexamethasone [87].

Various BiTE antibodies against EpCAM (Epithelial Adhesion molecule CD326), CEA (carcinoembryonic antigen) anti prostate specific membrane antigen, B7-H3 are being developed and may be a potential therapy option in solid malignancies [86].

### 7.2. Adoptive Cell Transfer (ACT)

The central premise in ACT is that T cells are crucial for eliminating cancer cells and hence transfer of T cells in expanded numbers can augment anti-tumor immunity. ACT involves isolating tumor infiltrating lymphocytes from cancers, growing them in culture and then reintroducing them to the patient who has undergone a lymphocyte depleting preparatory regimen [88]. In a cohort of 93 previously treated patients with metastatic melanoma who underwent ACT, the results were very encouraging. Fifty-six percent of patients had an objective response with 20 patients (22%) having a complete response [89]. All the complete responders, except one patient, had ongoing response beyond three years with a five-year overall survival rate of 93%. In metastatic solid tumors, cure is not thought to be possible except in select malignancies like germ cell tumors and hence ACT which has raised the specter of a cure is considered a very groundbreaking therapy. Indeed, ACT using HPV-tumor-infiltrating T cells has induced dramatic tumor regression in a cohort of cervical cancer patients [90]. However, the challenge is to do this in large scale in the clinic. Many innovations have been made recently including using peripheral blood to access T cells, obviating the need for resection of metastases. With modern genetic engineering technology, specific antigen receptors can be introduced into T cells allowing them to recognize tumor specific antigens [91]. These lymphocytes can then be produced on a large scale and used in patients. There are a couple of approaches for antigen receptor engineering. In one approach, a T cell receptor which is similar to the endogenous T cell receptor is introduced and these would still need activation through the antigen presenting cells/major histocompatibility complex. In the other approach, a chimeric antigen receptor (CAR) is introduced to the T lymphocyte surface with help of a viral vector [92]. CARs are specialized structures with both antigen binding as well as intracellular signaling apparatus such that they can recognize antigens independent of APCs and can also drive cellular activation [93]. CARs can be engineered with specificity to a chosen tumor specific antigen. These attributes can thus help override flaws in antigen presentation of cancers and also allow selective targeting of cancer. While the first generation CARs had a single chain variable fragment (scFv) attached to the transmembrane domain and T cell signaling unit without a co stimulatory domain, second and third generation CARs have been refined to include co stimulatory domains which have made them more effective [94]. CARs can auto signal leading to persistent activation causing potentially lethal cytokine release syndrome in addition to immunologic exhaustion. While the vast majority of ACT technology have focused on T cells, there is interest in expanding this to a variety of MHC unrestricted immune cells including NK cells, cytokine induced killer (CIK) cells and lymphokine activated killer cells (LAK) [95,96,97,98].

The most remarkable success story of CAR therapy has been in B cell malignancies including lymphomas, leukemias, CLL where a CD19 specific CAR has been used in chemotherapy refractory patients with impressive durable responses. In a report of 15 patients with advanced B cell malignancies undergoing anti CD 19 CAR therapy, there were eight CR and four PRs and one stable disease [99]. In addition to the cytokine release syndrome, another complication with anti CD 19 CAR therapy is B cell aplasia, which can be prolonged but was successfully managed with intravenous immunoglobulin infusions.

CAR therapy is also being investigated in solid tumors. Metastatic mesothelioma is regionally aggressive with improved prognosis for patients with higher levels of tumor infiltrating lymphocytes, highlighting the importance of cell-mediated anti-tumor response. Preclinical studies with adoptive cell transfer (ACT) of T cells expressing chimeric antigen receptors (CAR) against mesothelin (MSLN), a tumor antigen associated with decreased survival and overexpressed on the surfaces of more than 90% of epithelioid malignant pleural mesothelioma, resulted in enhanced T cell recruitment towards MSLN+ cells and a 2- to 5-fold increase in Th1 cytokine secretion. Importantly, studies with animal models showed that regional adoptive transfer of MSLN specific CAR T cells compared to systemic administration demonstrated enhanced T cells activation, anti-tumor efficacy, and duration of response. Based on these findings, phase I trials are currently being designed to determine the safety profile of CAR T cells for clinical use [100]. Nonetheless, one of the major hurdles particularly in solid tumors is that tumor-specific antigen usually can also be expressed by normal tissues leading to substantial toxicity. It has been proposed that by engineering antigen receptors against neoantigens solely expressed by an individual’s cancer cells can help avoid complications. However, this is time consuming as well as expensive and is not currently a practical approach in the clinic.

## 8. Conclusions

It is evident that there is tremendous growth in the field of Immune Oncology stemming from improved understanding of the interaction among cancer cells, TME and the body’s immune system. However, promising therapies like checkpoint inhibitors are effective only in a fraction of the patients. In order to augment responses, rational combinations of immunotherapeutic agents and new immunotherapy technologies are being vigorously investigated. Additionally, there is recognition that an individual’s cancer may exhibit private antigens or neoantigens that are very different from normal tissue antigens. These can form the basis for personalized immunotherapy strategies unique to a patient’s cancer with minimal side effects. The ultimate objective would be getting closer to the Holy Grail in cancer that is the development of curative therapy even in metastatic disease.

## Figures and Tables

**Table 1:** Examples of Immunotherapy agents and corresponding representative clinical trials in development.

Target	Mechanism	Experimental Agent	Oher Drugs/Intervention	Drug Administ Ration	Phase in Testing	Clinical Setting	Clinicaltrials. gov ID
Checkpoint Inhibitory Receptors	Anti-PD-1	STI-A1110	NA	IV mAb	preclinical	NA	
		PDR001	NA	IV mAB	Phase I/II	Solid tumors	NCT02404441
		MEDI0680	MEDI4736 (anti PD-l1)	IV mAB	Phase I/II	Advanced malignancies	NCT02118337
	Anti-PD-L1	LY3300054	Ramucirumab Neciitumumab	IV mAB	Phase I	Solid tumor	NCT02791334
		CA-170 (also targets PD-L2, VISTA)	NA	Oral	Phase I	Solid tumor Lymphoma	NCT02812875
		KN035	NA	IV mAB	Phase I	Solid Tumor	NCT02827968
	Anti-CTLA-4	AGEN1884		IV mAB	Phase I	Advanced malignancies	NCT02694822
		Tremelimumab	MEDI4736	IV mAB	Phase I-III	Head and neck	NCT02551159
	Anti-LAG3 (CD223)	LAG525	PDR001	IV mAB	Phase I/II	Advanced malignancies	NCT02460224
		BMS-986016	NA	IV mAB	Phase I/II	Hematological cancers	NCT02061761
	B7-H3 (CD276)	Enoblituzumab (MGA271)	NA	IV mAB	Phase I	Refractory ca	NCT01391143
		MGD009	NA	IV bispecific AB	Phase I	Metastatic cancers	NCT02628535
	Anti-VISTA	JNJ-61610588	NA	IV mAB	Phase I	Advanced malignancies	NCT02671955
	Anti-TIM3	MBG453	PDR001	IV mAB	Phase I/II	Advanced malignancies	NCT02608268
		TSR-022	Anti PD-1	IV mAB	Phase I	Advanced malignancies	NCT02817633
Checkpoint Activating Receptors	Ox40 (CD134) agonist	PF-04518600	4-1BB agonist	IV mAB	Phase I	Advanced malignancies	NCT02315066
		MEDI6469	NA	IV mAB (murine)	Phase I	Colorectal ca	NCT02559024
		MEDI0562	NA	IV mAB (humanized)	Phase I	Advanced malignancies	NCT02318394
		MEDI6383	MEDI4736	OX40 ligand fusion protein	Phase I	Advanced malignancies	NCT02221960
	4-1BB (CD137) agonist	Utomilumab (PF-05082566)	PF-05082566	IV mAB	Phase I	Advanced malignancies	NCT02315066
		Urelumab	Nivolumab	IV mAB	Phase I/II	Advanced	NCT02534506
	CD27	Varlilumab	Atezolizumab	IV mAB	Phase I/II	Advanced malignancies	NCT02543645
	GITR	GWN323	PDR001	IV mAb	Phase I	Advanced malignancies Lymphoma	NCT02740270
		TRX518	NA	IV mAB	Phase I	Melanoma] Solid cancers	NCT01239134
		INCAGN01876	NA	IV mAB	Phase I	Solid cancers	NCT02697591
		MK-4166	Pembrolizumab	IV mAB	Phase I	Solid cancers	NCT02132754
Intracellular or Extracellular Modulators of Immune Response in the TME	Anti-BTK	Ibrutinib	Durvalumab	Oral	Phase I	Soild cancers	NCT02403271
		Acalabrutinib	NA	Oral	Phase I/II	GBM	NCT02586857
	Anti-CSF-1R	LY3022855	Tremelimumab Durvalumab	IV mAB	Phase I	Solid cancers	NCT02718911
		MCS-110	PDR001	IV mAB	Phase I/II	Advanced malignancies	NCT02807844
		FPA008	Nivolumab	IV mAB	Phase I	Advanced malignancies	NCT02526017
		Pexidartinib	Durvalumab	Oral	Phase I	Pancreas ca Colorectal ca	NCT02777710
		BLZ945	PDR001	Oral	Phase I/II	Advanced malignancies	NCT02829723
		PLX3397	Pembrolizumab	Oral	Phase I/II	Melanoma Solid tumor	NCT02452424
	Adenosine A2A receptor antagonist	PBF-509	PDR001	Oral	Phase I	Lung cancer	NCT02403193
		CPI444	Atezolizumab	Oral	Phase I	Advanced malignancies	NCT02655822
	Anti-CCR4	Mogamulizumab (KW-061)	PF-05082566	IV mAB	Phase I	Advanced malignancies	NCT02444793
	Anti-KIR	BMS-986015	Ipilimumab	IV mAB	Phase I	Advanced ca	NCT01750580
		Lirilumab	Nivolumab Ipilimumab	IV mAB	Phase I	Multiple Myeloma	NCT01592370
	IDO1 inhibitor	GDC-0919	NA	Oral	Phase I	Solid cancers	NCT02048709
		Epacadostat (INCB024360)	NA	Oral	Phase I	Solid cancers	NCT02559492
		Indoximod	Docetaxel	Oral	Phase I	Lung cancer	NCT02460367
	JAK inhibitor	INCB039110	Pembrolizumab	Oral	Phase I	Advanced cancer	NCT02646748
